# Characterization of the complete mitochondrial genome of *Myrmuslateralis* (Heteroptera, Rhopalidae) and its implication for phylogenetic analyses

**DOI:** 10.3897/zookeys.1070.72742

**Published:** 2021-11-10

**Authors:** Wanqing Zhao, Dajun Liu, Qian Jia, Xin Wu, Hufang Zhang

**Affiliations:** 1 Department of Biology, Xinzhou Teachers University, Xinzhou 034000, Shanxi, China Xinzhou Teachers University Xinzhou China

**Keywords:** Mitogenome, next-generation sequencing, phylogeny, scentless plant bugs

## Abstract

Mitochondrial genomes (mitogenomes) are widely used in research studies on phylogenetic relationships and evolutionary history. Here, we sequenced and analyzed the mitogenome of the scentless plant bug *Myrmuslateralis* Hsiao, 1964 (Heteroptera, Rhopalidae). The complete 17,309 bp genome encoded 37 genes, including 13 protein-coding genes (PCGs), 22 transfer RNA (tRNA) genes, two ribosomal RNA (rRNA) genes, and a control region. The mitogenome revealed a high A+T content (75.8%), a positive AT-skew (0.092), and a negative GC-skew (–0.165). All 13 PCGs were found to start with ATN codons, except for *cox1*, in which TTG was the start codon. The Ka/Ks ratios of 13 PCGs were all lower than 1, indicating that purifying selection evolved in these genes. All tRNAs could be folded into the typical cloverleaf secondary structure, except for *trnS1* and *trnV*, which lack dihydrouridine arms. Phylogenetic trees were constructed and analyzed based on the PCG+rRNA from 38 mitogenomes, using maximum likelihood and Bayesian inference methods, showed that *M.lateralis* and *Chorosomamacilentum* Stål, 1858 grouped together in the tribe Chorosomatini. In addition, Coreoidea and Pyrrhocoroidea were sister groups among the superfamilies of Trichophora, and Rhopalidae was a sister group to Alydidae + Coreidae.

## Introduction

Mitochondria are important cytoplasmic organelles in eukaryotic cells, playing a critical role in cell metabolism, disease, apoptosis, and senescence. Mitochondrial genomes are characterized by a simple structure, stable composition, conserved arrangement, maternal inheritance, and rapid evolutionary rate (Simon et al. 2006; Cameron 2014; Tyagi et al. 2020). Therefore, mitochondrial genomes (mitogenomes) have been widely used in phylogenetic analyses and studies on population genetics (Yuan et al. 2015; Song et al. 2019; Xu et al. 2021). For most insects, the mitogenome is typically a double-stranded circular DNA molecule from 15 to 20 kb in size, including 13 protein-coding genes (PCGs), 22 transfer RNA (tRNA) genes, two ribosomal RNA (rRNA) genes, as well as a long non-coding region, also known as the control or AT-rich region (Wolstenholme 1992; Boore 1999; da Fonseca et al. 2008; Yang et al. 2018). Next-generation sequencing enables large amount of sequence data to be obtained and analyzed in a more economically efficient manner. As a result of advances in this technology, it is now more feasible to obtain the complete mitogenome of large taxa; thus, molecular phylogenetic analysis has been revolutionized (Beckenbach et al. 2009; Kocher et al. 2014).

Rhopalidae, commonly known as scentless plant bugs, is a family of Pentatomomorpha insects in the superfamily Coreoidea (Schaefer and Chopra 1982; Schaefer and Panizzi 2000; Steill and Meyer 2003). The Rhopalidae includes 26 genera and 279 species distributed throughout the biogeographical areas of the world (CoreoideaSF Team 2021); many species are of critical importance as pests of pasture grass (Carroll and Loye 2012). In China, Rhopalidae includes two subfamilies, Serinethinae (containing only one genus) and Rhopalinae (containing 20 genera) (Dolling 2006). Phylogeny for the tribes of Rhopalinae has been hypothesized based on morphology, but several concurrent hypotheses still exist (Schaefer 1993; Li and Zheng 1994; Davidova-Vilimova et al. 2000; Henry 2019). Although there are several published studies examining the relationships among infrafamilial levels of the Coreoidea, a consensus regarding the relationships among different families and subfamily lineages has not been reached (Souza et al. 2009; Ghahari et al. 2012; Forthman et al. 2019). In addition, some studies support Aradoidea as sister group of Trichophora (which includes Pentatomoidea, Lygaeoidea, Pyrrhocoroidea and Coreoidea), but the relationship within Trichophora is still under debate (Li et al. 2005; Xie et al. 2005; Linnavuori 2007; Tian et al. 2011; Weirauch and Schuh 2011).

*Myrmuslateralis* Hsiao, 1964 is an endemic species of China (Beijing, Hebei, eastern Inner Mongolia), Korea, Mongolia, and Russia (Far East) (Liu et al. 1994; Aukema and Rieger 2006; CoreoideaSF Team 2021). Adults are present in both forests and grasslands during July and August (Nonnaizab 1986). To date, only four complete mitogenomes from the Rhopalidae have been published in GenBank, and all of them belong to Rhopalinae (Table 1). According to the latest classification, five tribes are classified in the Rhopalidae (CoreoideaSF Team 2021). The tribe Chorosomatini Fieber, 1860 contains five genera (*Agraphopus* Stål, 1872; *Chorosoma* Curtis, 1830; *Ithamar* Kirkaldy, 1902; *Leptoceraea* Jakovlev, 1873; *Myrmus* Hahn, 1832 and *Xenogenus* Berg, 1883), of which only *Myrmus* and *Chorosoma* are distributed in China.

**Table 1.** List of sequences used to reconstruct the phylogenetic tree.

	Family	Species	Accession number
**Outgroup**	Nabidae	*Himacerusnodipes*	JF927832
**Ingroup**	Aradidae	*Aradacanthiaheissi*	HQ441233
		*Araduscompar*	NC_030362
		*Aneurussimilis*	NC_030360
	Cydnidae	*Macroscytusgibbulus*	NC_012457
		*Adrisamagna*	NC_042429
		*Scoparipessalvazai*	MF614955
	Pentatomidae	*Eurydemadominulus*	NC_044762
		*Palomenaviridissima*	NC_050166
		*Eysarcorisguttigerus*	MN831205
		*Armacustos*	MT535604
		*Pentatomasemiannulata*	MT985377
	Colobathristidae	*Phaenacanthamarcida*	EU427342
	Rhyparochromidae	*Panaorusalbomaculatus*	NC_031364
	Malcidae	*Malcusinconspicuus*	EU427339
	Lygaeidae	*Kleidocerysresedae*	KJ584365
	Pyrrhocoridae	*Dindymusrubiginosus*	NC_042439
		*Euscopusrufipes*	NC_042436
		*Melamphausfaber*	NC_042435
		*Dysdercusdecussatus*	NC_042438
		*Antilochuscoquebertii*	NC_042441
	Rhopalidae	*Stictopleurussubviridis*	EU826088
		***Myrmuslateralis***	**MN412595**
		*Chorosomamacilentum*	MN412594
		*Aeschyntelusnotatus*	EU427333
		*Corizus* sp.	KM983397
	Alydidae	*Riptortuspedestris*	EU427344
		*Neomegalotomusparvus*	MG253274
	Coreidae	*Hydaropsislongirostris*	EU427337
		*Clavigrallatomentosicollis*	KY274846
		*Acanthocoris* sp.	MF497707
		*Enoplopspotanini*	MF497720
		*Leptoglossusmembranaceus*	MF497724
		*Anoplocnemiscurvipes*	KY906099
		*Mictistenebrosa*	MF497729
		*Pseudomictisbrevicornis*	MF497732
		*Molipteryxlunata*	MF497721
		*Notopteryxsoror*	KX505857

In this study, we sequenced and annotated the complete mitogenome of *M.lateralis*. We examined the genomic features, nucleotide composition, codon usage, RNA secondary structure, evolutionary pattern of 13 PCGs and characteristics of the control region. Finally, we evaluated the phylogenetic relationship of the mitochondrial sequence data at different taxonomic levels.

## Material and methods

### Sampling, DNA extraction and sequencing

Adult specimens of *Myrmuslateralis* were collected from Jincheng City, Shanxi Province, China on 20 July 2014. Specimens were preserved in 100% ethanol and stored at –20 °C. The genomic DNA was extracted from leg muscles using a ONE-4-ALL Genomic DNA Mini-Prep Kit (BS88504; Sangon, Shanghai, China) according to the manufacturer’s protocols. The mitogenome was sequenced using the whole-genome shotgun method on an Illumina Miseq platform (Personalbio, Shanghai, China). After filtering low-quality and adapter contaminated reads, A5-miseq version 20150522 (Coil et al. 2015) was used for contig assembly.

### Gene annotation and sequence analysis

Sequence annotation was performed using Geneious 10.1.3 (Kearse et al. 2012) and the MITOS web server (Bernt et al. 2013). Annotations of 13 protein-coding regions were edited manually by predicting open reading frames using the invertebrate mitochondrial code. The secondary structures of the tRNA genes were predicted with tRNAscan-SE web server (http://lowelab.ucsc.edu/tRNAscan-SE/) (Tamura et al. 2013). The identification of rRNA genes was performed based on their putative secondary structures and by comparing nucleotide sequences with those of previously reported mitogenomes. The control region was identified through the boundary of neighboring genes. The number of tandem repeats of control region was investigated with Tandem Repeats Finder (http://tandem.bu.edu/trf/trf.html) (Benson 1999) and the presence of stem-loop was predicted by Mfold Web Server (http://mfold.rna.albany.edu) (Zuker 2003). The annotated mitogenome sequence of *Myrmuslateralis* has been submitted to GenBank under the accession number MN412595.

The base compositions, codon usages, and relative synonymous codon usage (RSCU) values were calculated by MEGA 6.0. AT and GC skew were calculated by the following formulae: AT skew = (A–T)/(A+T) and GC skew=(G–C)/(G+C). To analyse the evolutionary patterns of the 13 PCGs, the rates of nonsynonymous substitution (Ka), the rates of synonymous substitution (Ks), and the ratio of Ka/Ks for each gene were calculated by the software DnaSP 5.0 (Librado and Rozas 2009).

### Phylogenetic analyses

In addition to the mitogenome newly sequenced here, 37 other mitogenomes were taken from Genbank for the phylogenetic analyses (Table 1). The nucleic acids of 13 PCGs and two rRNAs were extracted by Geneious 10.1.3 and aligned with the Muscle algorithm in MEGA 6.0. Finally, the individual alignments were concatenated to make the datasets of PCG+rRNA (nucleotide alignment including 13 protein-coding genes and two rRNA genes) with SequenceMatrix (Vaidya et al. 2011).

The dataset was used to reconstruct phylogenetic trees under Bayesian inference (BI) and maximum likelihood (ML) using MrBayes 3.2.6 (Ronquist et al. 2012) and RaxML 8.0.2 (Stamatakis 2015) respectively. BI analysis was performed using a TVM+I+G model selected by jModeltest (Darriba et al. 2012). Two independent runs of four Markov Chains Monte Carlo (MCMC) chains in parallel for 5,000,000 generations were applied and sampled every 1,000 generations. Tracer 1.7 (Rambaut et al. 2018) was used to analyze the trace files from the Bayesian MCMC runs. Stationarity was considered to be reached when ESS (estimated sample size) value above 100 as MrBayes suggested. The first 25% of sampled trees were discarded as burn-in, and the remaining trees were used to calculate a 50% majority rule consensus tree. The ML tree was constructed using the GTR+I+G model, and nodal support values were evaluated through an ultrafast bootstrap approach, with 10,000 replicates.

## Results

### Genome organization

The 17,309 bp mitochondrial genome of *Myrmuslateralis* was determined to be a typical circular nucleotide molecule, consisting of 13 PCGs, 22 tRNA genes, two rRNA genes, and a putative control region (Table 2). For the gene arrangement, 23 genes (nine PCGs and 14 tRNAs) were found on the majority strand and 14 genes (four PCGs, eight tRNAs, and two rRNAs) were found on the minority strand. In addition, the mitogenome contains 10 gene overlaps totaling 37 bp and ranging from 1 to 8 bp; the longest overlap is being found between *trnW* and *trnC*. Thirteen intergenic spacers equal to 77 bp were observed, ranging from 1 to 18 bp, the longest being found between *trnS2* and *nad1*.

**Table 2.** Organization of the mitochondrial genome of *Myrmuslateralis*.

Gene	Strand	Size(bp)	Position		Intergenicnucleotides(IGN)	Anticodon	Codons	
			Start	Stop			Start	Stop
*trnI*	J	66	1	66	0	GAU	—	—
*trnQ*	N	69	64	132	2	UUG	—	—
*trnM*	J	69	137	205	4	CAU	—	—
*nad2*	J	985	207	1191	1	—	ATG	T
*trnW*	J	63	1204	1266	12	UCA	—	—
*trnC*	N	62	1259	1320	−8	GCA	—	—
*trnY*	N	64	1321	1384	0	GUA	—	—
*cox1*	J	1539	1387	2925	2	—	TTG	TAA
*trnL2^UUR^*	J	66	2921	2986	−5	UAA	—	—
*cox2*	J	676	2987	3662	0	—	ATT	T
*trnK*	J	72	3663	3734	0	CUU	—	—
*trnD*	J	65	3738	3802	3	GUC	—	—
*atp8*	J	162	3803	3964	0	—	ATA	TAA
*atp6*	J	669	3958	4626	−7	—	ATG	TAA
*cox3*	J	790	4626	5415	−1	—	ATG	T
*trnG*	J	63	5413	5475	−3	UCC	—	—
*nad3*	J	349	5476	5824	0	—	ATA	T
*trnA*	J	63	5825	5887	9	UGC	—	—
*trnR*	J	64	5891	5954	3	UCG	—	—
*trnN*	J	66	5954	6019	−1	GUU	—	—
*trnS1^AGN^*	J	69	6023	6091	3	GCU	—	—
*trnE*	J	64	6091	6154	−1	UUC	—	—
*trnF*	N	66	6154	6219	−1	GAA	—	—
*nad5*	N	1702	6220	7921	0	—	ATA	T
*trnH*	N	63	7931	7993	9	GUG	—	—
*nad4*	N	1317	7994	9310	0	—	ATG	TAA
*nad4L*	N	288	9304	9591	−7	—	ATT	TAA
*trnT*	J	62	9594	9655	3	UGU	—	—
*trnP*	N	64	9656	9719	0	UGG	—	—
*nad6*	J	483	9728	10210	8	—	ATA	TAA
*cytB*	J	1137	10210	11346	−1	—	ATG	TAG
*trnS2^UCN^*	J	69	11345	11413	−2	UGA	—	—
*nad1*	N	927	11432	12358	18	—	ATT	TAA
*trnL1^CUN^*	N	67	12359	12425	0	UAG	—	—
*rrnL*	N	1261	12426	13686	0	—	—	—
*trnV*	N	69	13687	13755	0	UAC	—	—
*rrnS*	N	967	13756	14722	0	—	—	—
Control region		2587	14723	17309	0	—	—	—

Note: J refers to major strand; N refers to minus strand

### Nucleotide composition and codon usage

The mitochondrial genome of *M.lateralis* was strongly biased toward A+T (75.8%) in nucleotide composition, with 75.5%, 75.7%, 71.3%, and 74.8% A+T content in PCGs, rRNAs, tRNAs, and control regions, respectively. The nucleotide composition and skewness of the mitogenome is shown in Table 3. The three codon positions of PCGs possessed different A+T content; the third site had the highest value (80.1%), whereas the second site had the lowest (74.3%). The mitogenome had more A and G content than T and C, with a positive AT-skew (0.092) and a negative GC-skew (–0.165). The AT-skew of PCGs and three codon positions were all negative, –0.018 in PCGs, –0.214 in the first, –0.050 in the second, and –0.099 in the third codon positions. The GC-skew of PCGs (0.033) and the second codon position (0.127) was slightly and moderately positive, respectfully, while the first and third codon positions were both negative (–0.018 and –0.020, respectively). AT-skew values for the tRNA and rRNA genes were both negative (–0.057 and –0.001, respectively), while the GC-skew was obviously positive for tRNAs (0.292) and slightly positive for rRNAs (0.019).

**Table 3.** Nucleotide composition and skewness of the *Myrmuslateralis* mitochondrial genome.

**Feature**	**T**%	**C**%	**A**%	**G**%	**A+T**%	**AT-skew**	**GC-skew**
Whole genome	34.4	14.1	41.4	10.1	75.8	0.092	−0.165
PCGs	42.2	11.8	33.3	12.6	75.5	−0.118	0.033
PCGs-1^st^	44	14.1	28.5	13.6	72.5	−0.214	−0.018
PCGs-2^nd^	39	11	35.3	14.2	74.3	−0.05	0.127
PCGs-3^rd^	44	10.4	36.1	10	80.1	−0.099	−0.02
tRNAs	40	8.6	35.7	15.7	75.7	−0.057	0.292
rRNAs	35.7	14.1	35.6	14.7	71.3	−0.001	0.019
Control region	35	15.9	39.8	9.4	74.8	0.064	−0.257

**Figure 1.**
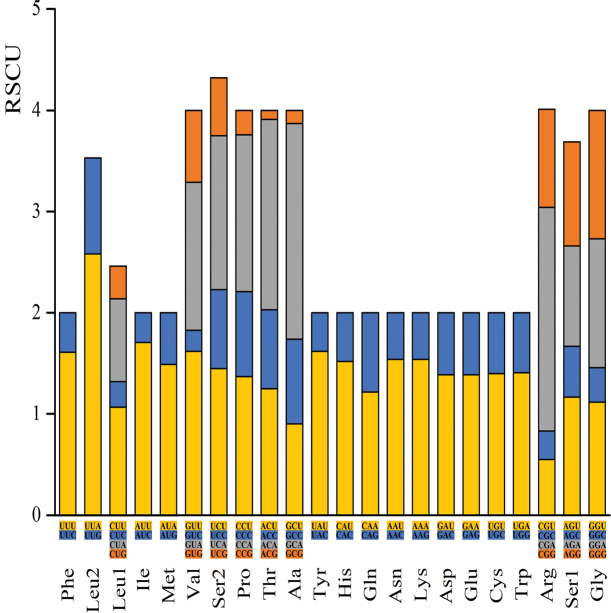
The relative synonymous codon usage (RSCU) in the *Myrmuslateralis* mitogenome.

Relative synonymous codon usage (RSCU) values of the 13 PCGs were calculated based on 3633 codons (Fig. 1). UUU (F, Phe), UAU (Y, Tyr), UUA (L, Leu2), and AUU (I, Ile) were the most frequently used codons, accounting for 28.24% of all codons. All synonymous codons ending with A or U were more frequent than those ending with C or G, except in three cases: CGG (RSCU = 0.97) was used more than CGU (RSCU = 0.55) for Arg, AGG (RSCU=1.03) was used more than AGA (RSCU = 0.99) for Ser1, and GGG (RSCU=1.27) was used more than GGU (RSCU = 1.12) for Gly.

### Protein coding genes

In the *M.lateralis* mitogenome, nine PCGs were located on the J-strand (majority strand) and four PCGs were located on the N-strand (minority strand). The PCGs had a total length of 11,024 bp that accounted for 63.69% of the complete mitogenome. Among the mitochondrial proteins, Leu (16.67%), Phe (11.02%), and Tyr (9.58%) were the most frequent amino acids.

Most PCGs started with a typical ATN codon; five started with ATG (*nad2, atp6, cox3, nad4*, and *cytb*), four with ATA (*atp8, nad3, nad5*, and *nad6*), and three with ATT (*cox2, nad4L, and nad1*). The only unusual initiation codon was TTG in *cox1*. Among the 13 PCGs, seven genes ended with the complete stop codon TAA (*cox1, atp8, atp6, nad1, nad4*, and *nad4L*) or TAG (*cytb*), whereas five genes ended with the partial termination codon T (*nad2, cox2, cox3, nad3*, and *nad5*).

**Figure 2.**
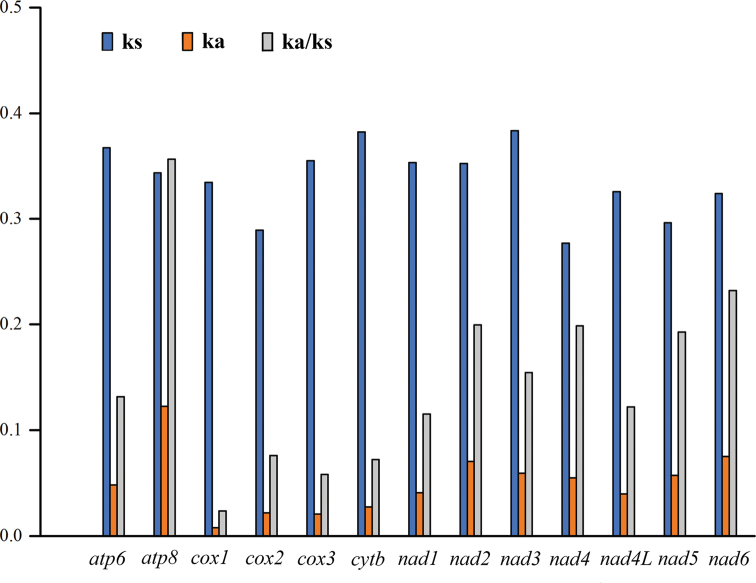
The rates of nonsynonymous substitution (Ka), the rates of synonymous substitution (Ks), and the ratio of Ka/Ks for each PCGs of *Myrmuslateralis* mitogenome

The evolutionary patterns of the 13 PCGs were analyzed and shown in Figure 2. The highest values for both Ka and Ks were observed for *atp8 and cytb*. *atp8* exhibited the highest Ka/Ks value, whereas *cox1* exhibited the lowest. The Ka/Ks values for all 13 PCGs were below 0.29.

### Transfer RNAs and ribosomal RNAs

**Figure 3.**
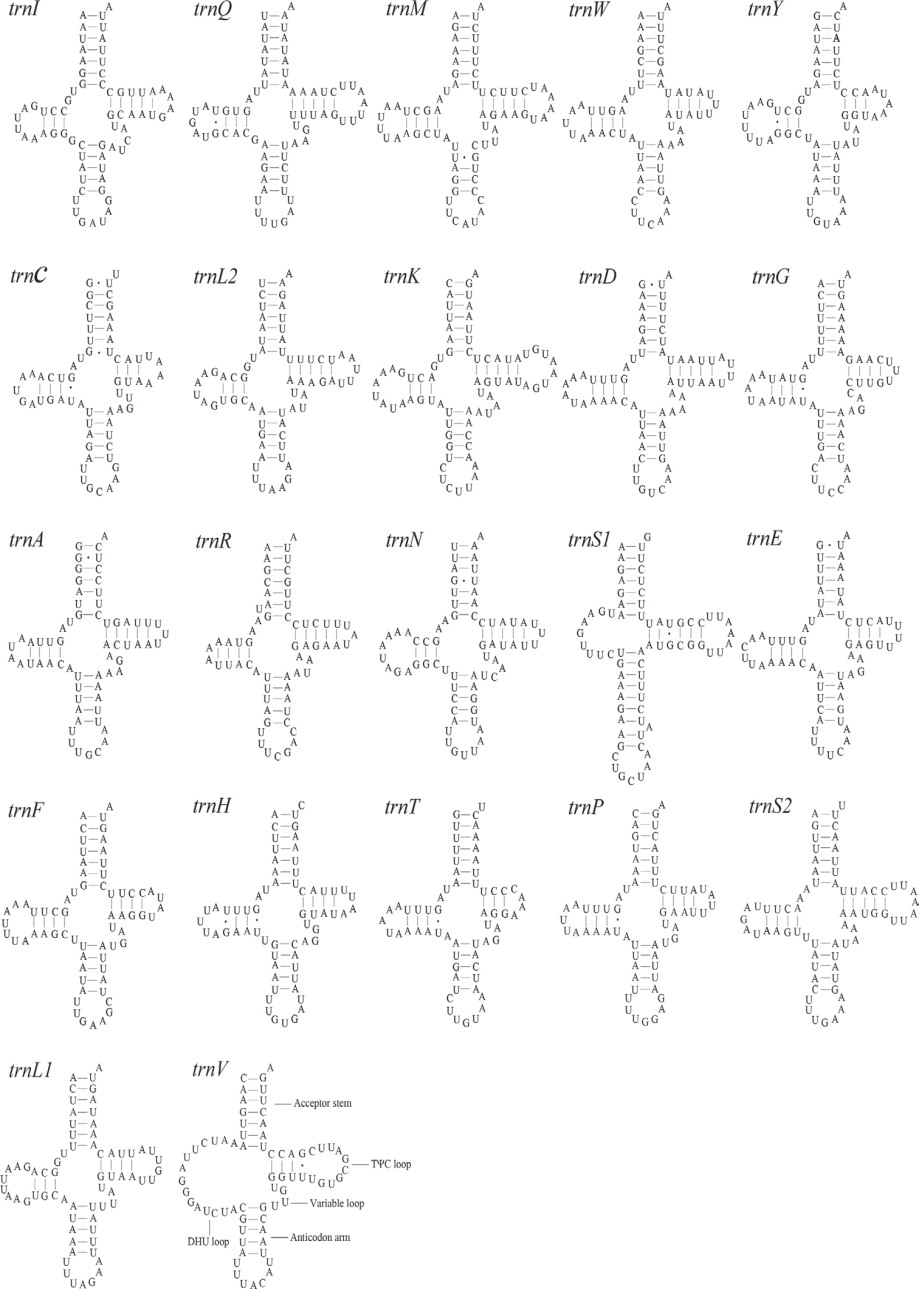
Predicted secondary structure of tRNA genes in the *Myrmuslateralis* mitogenome.

A total of 22 tRNA genes were encoded by the *M.lateralis* mitogenome, ranging from 62 bp (*trnC* and *trnT*) to 72 bp (*trnK*) in length (Table 2). Eight tRNA genes (*trnQ, trnC, trnY, trnF, trnH, trnP, trnL1^(CUN)^* and *trnV*) were encoded on the N-strand; the remaining 14 genes were encoded on the J-strand. The 22 tRNA genes had a total length of 1,445 bp, accounting for 8.34% of the complete mitogenome. The predicted secondary structures are shown in Figure 3. All tRNA genes could be folded into typical cloverleaf secondary structures, except for *trnS1* and *trnV*, in which the necessary dihydrouridine (DHU) arms were replaced with a simple loop. A total of 16 wobbled G-U pairs were found (six in acceptor stems, eight in DHU stems, one anticodon stems, and one in TΨC stems) that formed weak bonds in the tRNAs.

**Figure 4.**
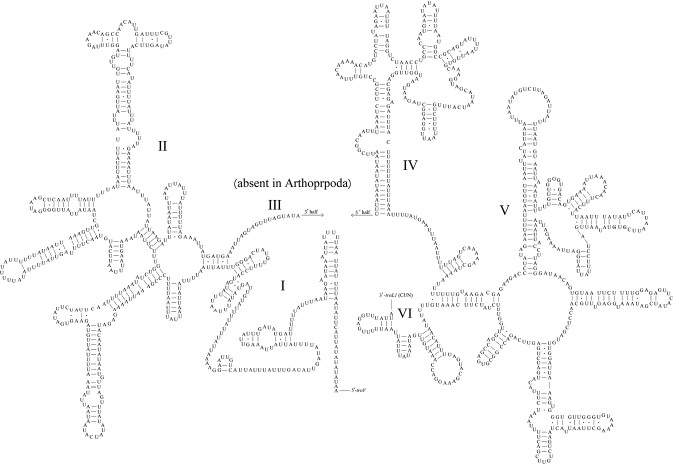
Predicted secondary structure of the *rrnL* in the *Myrmuslateralis* mitogenome.

**Figure 5.**
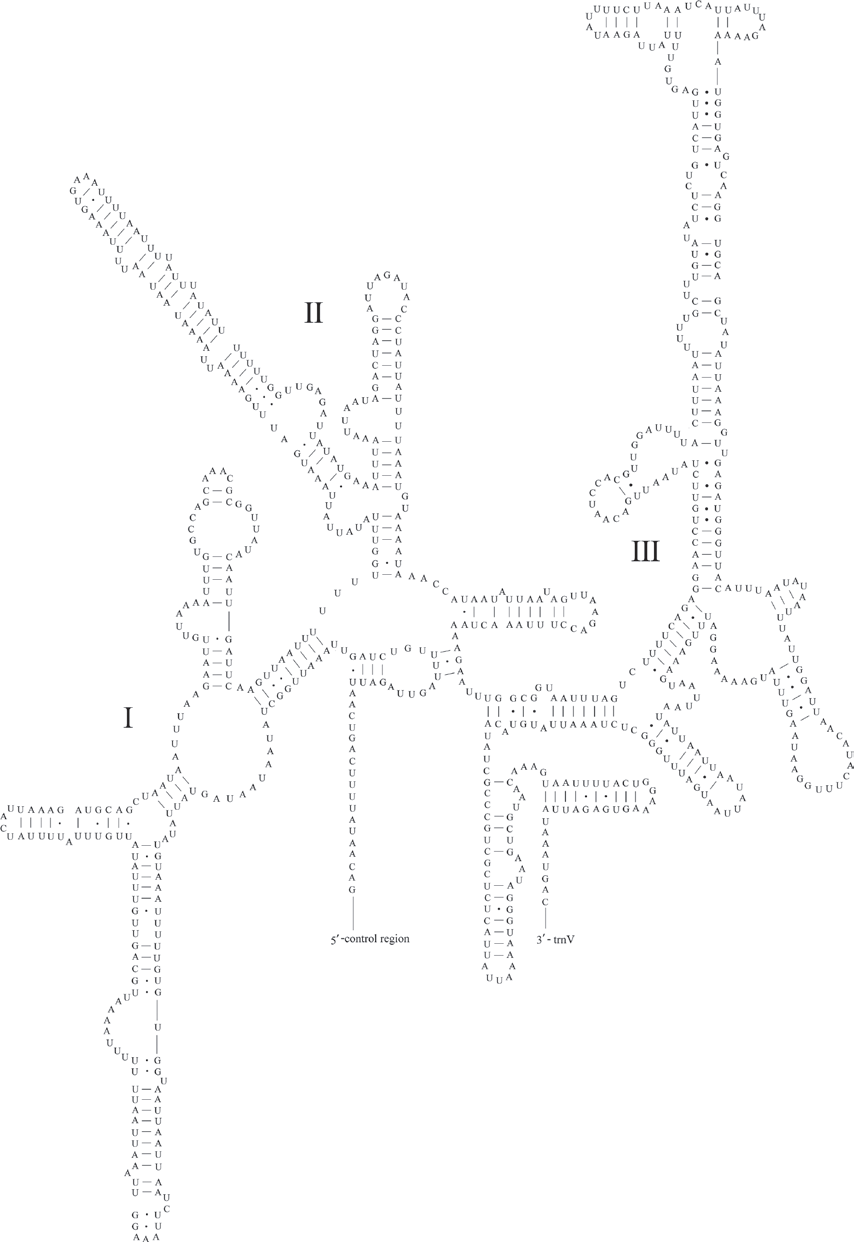
Predicted secondary structure of the *rrnS* in the *Myrmuslateralis* mitogenome.

The large 1,261 bp *rrnL* gene was located between *trnL1 ^(CUN)^* and *trnV*, and the small 967 bp *rrnS* gene was located between *trnV* and the control region. The secondary structures of both the *rrnL* and *rrnS* genes are shown in Figures 4, 5. The secondary structure of the *rrnL* gene contained five domains (I, II, IV, V, VI); domain III was absent, whereas the *rrnS* gene consisted of three domains (I, II, III). Both rRNAs included many mismatched base pairs, most of which were G-U.

### Control region

**Figure 6.**
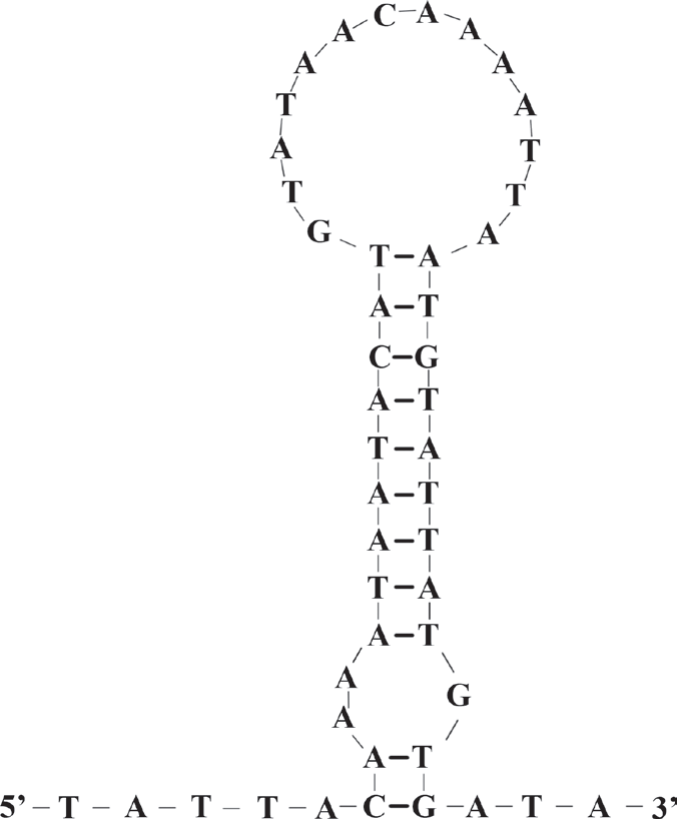
The stem-loop of control region in the *Myrmuslateralis* mitogenome.

The 2587 bp mitochondrial control region of *M.lateralis* was located between the *rrnS* gene and *trnI.* The A+T content (74.76%) was higher than the G+C content (25.24%), with a positive AT-skew and a negative GC-skew. In the control region, tandem repeat sequences, polyT stretch, polyA stretch, stem-loop structure, tandem repeats, and G(A)_n_ motif were commonly found. Based on these features, we identified the following important elements: a 13 bp polyT stretch, a G(A)_9_T sequence, an ATAGA motif, and a 9 bp polyA stretch. In addition, a stem-loop structure was observed at the end of the control region (Fig. 6). Although no longer tandem repeat sequences were identified, stretches of T(A)_4_ occurred many times.

### Phylogenetic analyses

**Figure 7.**
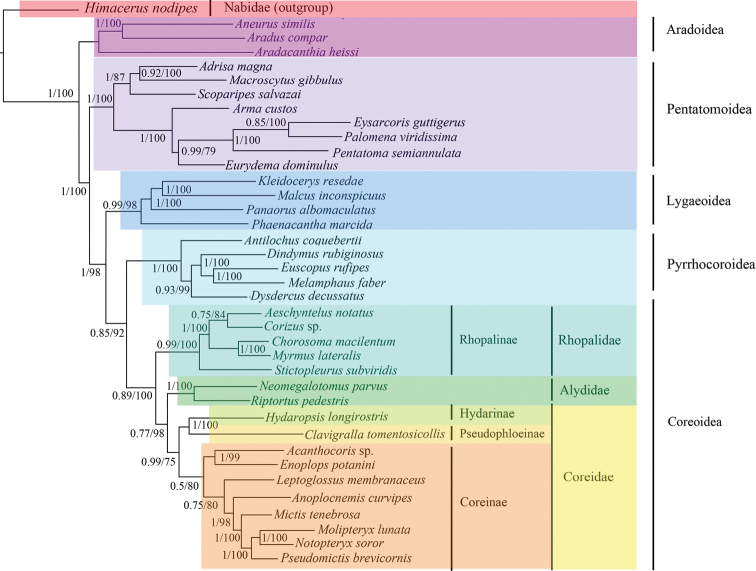
The phylogenetic relationships of PCG+rRNA using BI and ML methods. Numbers above each node indicate Bayesian posterior probabilities values and ML bootstrap values.

We conducted phylogenetic analyses based on the nucleotide sequences of the PCG+rRNA from eleven families within five superfamilies; one species from the Nabidae (damsel bugs) was used as an outgroup. The dataset contained 14,128 nucleotide sites from these 38 taxa. The BI and ML analyses generated identical phylogenetic results with most posterior probabilities (PP) of one and bootstrap pseudoreplicates (BP) of 100 (Fig. 7). Pentatomoidea, Lygaeoidea, Pyrrhocoroidea, and Coreoidea, the trichophorans, were all monophyletic and highly supported in both analyses (PP > 0.85 and BP > 92). A sister relationship between Coreoidea and Pyrrhocoroidea was recovered, and Pentatomoidea formed a sister group with the other superfamilies of the remaining Trichophora. Both analyses provided robust support (PP > 0.95 and BP > 85) for three families within Coreoidea: Alydidae was closer to Coreidae than Rhopalidae. Subfamilies of Coreidae were also supported with an internal relationship of ((Hydarinae + Pseudophloeinae) + Coreinae) (PP > 0.77 and BP > 75). Among Rhopalinae species, *Stictopleurussubviridis* Hsiao, 1977 was identified as sister to a group of other Rhopalinae species (PP = 0.99 and BP = 100). *M.lateralis* was close to *Chorosomamacilentum* Stål, 1858 (PP = 1 and BP = 100) and they both belonged to the tribe Chorosomatini.

## Discussion

In this study, we sequenced the mitogenome of *Myrmuslateralis* and built the molecular phylogenetic relationships with 37 other heteropteran taxa. The mitogenome was found to be 17,309 bp long, with 37 genes arranged consistent with other species of Hemiptera (Song et al. 2012; Li et al. 2017; Zhao et al. 2021). The control region represented the largest reported non-coding region for insect mitogenomes owing to its various motifs and tandem repeats (Cook et al. 1999; Li et al. 2015). Although the 2587 bp long control region possessed several elements, such as a polyT stretch, a G(A)_n_T sequence, an ATAGA motif, a stem-loop, and a polyA stretch, tandem repeats were not observed.

The present study analyzed the initiation and termination codons of the 13 PCGs. The majority of PCGs used common start codons (ATN), except for *cox1*, which was initiated with TTG, a frequently used start codon in heteropteran mitogenomes (Quek et al. 2021; Zhao et al. 2021). The partial termination codon T was found in *nad2, cox2, cox3, nad3* and *nad5*. Some researchers have proposed that the incomplete termination codon could be completed via post-transcriptional polyadenylation (Ojala et al. 1981). Among the synonymous codons, codons ending with an A or U were more frequent than those ending in a G or C and were also observed in other heteropteran species (Zhao et al. 2019; Li et al. 2021). In this study, the lowest evolutionary rate was observed in *cox1*, indicating that this gene can be effectively used as a DNA barcoding marker. The Ka/Ks values for all 13 PCGs were far less than one, indicating that all of the PCGs evolved under purifying selection, thus, they can be used to investigate phylogenetic relationships.

Although many studies have reconstructed phylogenetic relationships among members of Pentatomomorpha, the relationships of different levels are still ambiguous (Li et al. 2005; Liu et al. 2019; Souza-Firmino et al. 2020; Kaur and Singh 2021). The results from previous research were congruent with the result that Trichophora includes four superfamilies of Pentatomomorpha except Aradoidea, and with the monophyly of four superfamilies of Trichophora (Henry 1997; Hua et al. 2008; Yuan et al. 2015; Song et al. 2019). Hua et al. (2008) and Liu et al. (2019) supported Pentatomoidea as the basal group of Trichophora and Coreoidea as a sister group to Lygaeoidea based on mitochondrial genomes. The superfamilies Coreoidea and Pyrrhocoroidea were supported as sister taxa based on cladistic analysis (Henry 1997), as well as 37 mitochondrial genes analysis (Yuan et al. 2015). Whereas our mitogenomic data showed that Coreoidea and Pyrrhocoroidea were sister groups based on ML and BI analyses using the PCG+rRNA dataset. This result from our study was consistent with that from the study of Song et al. (2019) based on relationship analysis of PentatomomorphaPCGs.

At the family level, Rhopalidae was found to be a sister group to Alydidae + Coreidae. This relationship, based on mitogenomes, was consistent with that based on cladistic analysis by Li (1996) and Henry (1997). Despite the availability of numerous species, the classification patterns for the family Coreidae were weak in terms of both morphology and molecular data (Bressa et al. 2008; Forthman et al. 2019; Froeschner 2019). In this study, Hydarinae and Pseudophloeinae grouped the sister cluster. However, due to the limited samples, the relationships among Coreidae needs more studies in the future.

Previous studies on the classification of Rhopalinae came to a variety of conclusions and the positions of some species were not clear (Schaefer and Chopra 1982; Liu 1994; Aukema and Rieger 2006). The hypothesis that the genus *Stictopleurus* belongs to the Rhopalini tribe was corroborated based on morphological studies by Aukema and Rieger (2006). However, it did not group together with other Rhopalini species in this study. The monophyly of Rhopalini should be explored in the future studies with additional specimens.

## Conclusions

The complete mitogenome of the scentless plant bug *Myrmuslateralis* was sequenced using next-generation sequencing technologies, providing the fifth mitogenome sequence from approximately 230 species of Rhopalidae. The nucleotide composition, codon usage, RNA structures, and protein-coding genes evolution were analyzed in our paper. The mitogenome of *M.lateralis* revealed the phylogenetic position of *Myrmus*. However, more mitogenomes should be sequenced to investigate the mitogenomic evolution and phylogenetic relationships of Rhopalidae.
